# Generic escitalopram initiation and substitution among Medicare beneficiaries: A new user cohort study

**DOI:** 10.1371/journal.pone.0232226

**Published:** 2020-04-30

**Authors:** Chao Li, Li Chen, Nan Huo, Ahmed Ullah Mishuk, Richard A. Hansen, Ilene Harris, Zippora Kiptanui, Zhong Wang, Jingjing Qian

**Affiliations:** 1 Auburn University Harrison School of Pharmacy, Auburn, AL, United States of America; 2 Indiana University School of Medicine, Indianapolis, IN, United States of America; 3 Mayo Clinic, Rochester, MN, United States of America; 4 IMPAQ International LLC, Columbia, MD, United States of America; 5 Office of Research and Standards, Office of Generic Drugs, U.S. Food and Drug Administration, Center for Drug Evaluation and Research, Silver Spring, MD, United States of America; University of South Carolina College of Pharmacy, UNITED STATES

## Abstract

**Objectives:**

To examine patterns of generic escitalopram initiation and substitution among Medicare beneficiaries.

**Methods:**

This retrospective new user cohort used a 5% random sample of 2013–2015 Medicare administrative claims data. Fee-for-service Medicare beneficiaries continuously enrolled in Parts A, B, and D during a 6-month washout period prior to their initial generic or brand oral escitalopram prescriptions were included (n = 12,351). The primary outcomes were generic escitalopram treatment initiation, and among brand escitalopram initiators, generic substitution within 12 months. Patient demographics, health service utilization, and prescription level factors were measured and assessed.

**Results:**

Among all escitalopram initiators, about 88.2% Medicare beneficiaries initiated generic escitalopram. Beneficiaries who were younger age, male, residing in non-Northeast regions or urban area, in the Part D plan deductible benefit phase, and filling prescriptions at community/retail pharmacies were more likely to initiate generic treatment. Among brand escitalopram initiators (n = 1,464), about 20.7% switched to generic escitalopram, 31.2% switched to another alternative antidepressant, 25.1% discontinued treatment, and 8.7% were lost to follow up or passed away within 12 months after brand initiation. Factors associated with generic escitalopram substitution included region (Midwest vs. Northeast, adjusted hazard ratio (HR) = 1.46, 95% CI = 1.04–2.05), pre-index hospitalization (HR = 1.31; 95% CI = 1.16–1.48) and lower escitalopram average daily dosage (HR = 0.97; 95% CI = 0.95–0.99).

**Conclusions:**

In 2013–2015, almost 90% Medicare beneficiaries initiated generic escitalopram treatment. Among brand escitalopram initiators, about 1 in 5 patients switched to generic escitalopram within 1 year, as compared to 1 in 4 or 1 in 3 who discontinued current or switched to alternative treatment, respectively. Medicare beneficiary’s geographic region was independently associated with generic escitalopram initiation and substitution. Findings from this study not only provide up-to-date evidence in generic escitalopram use patterns among Medicare population, but also can guide educational and practice interventions to further increase generic escitalopram use.

## Introduction

Antidepressants are one of the three most commonly prescribed therapeutic drug classes in the U.S.[1, 2]. The most recent estimates of antidepressant use among noninstitutionalized U.S. population indicated that 12.7% of individuals aged 12 and above took antidepressant medications, and one-fourth of them had been on treatments for 10 years or longer [1]. Selective serotonin reuptake inhibitors (SSRIs) have increasingly become the first choice of antidepressant treatments for major depressive disorder and generalized anxiety disorder due to better efficacy and tolerability [3, 4]. However, antidepressant treatments are often accompanied by premature discontinuation and switching of treatments [5], which may be due to patient’s economic burden [6, 7].

Escitalopram is one of the most commonly used SSRIs among Medicare beneficiaries [8]. Although escitalopram has shown better acceptability and fewer discontinuations than other antidepressants (such as duloxetine, fluvoxamine, paroxetine, reboxetine, and venlafaxine) [7, 9], patients treated with brand escitalopram had significantly higher prescription costs and worse adherence in the past compared to those using other SSRIs such as citalopram and sertraline [8]. In March 2012, the U.S. Food and Drug Administration (FDA) approved the first generic escitalopram [10]. The FDA reviews and approves generic drugs through abbreviated new drug application (ANDA) based on pharmacological equivalence and bioequivalence testing, and approved generics should perform the same as their corresponding Reference Listed Drugs (RLD) [11].

The economic savings generated from the use of generic drugs are significant, totaling $265 billion in 2017 alone [12]. In addition, increasing generic drug use can reduce patients’ prescription costs [13, 14], improve medication adherence and promote health outcomes [6, 15]. Understanding generic escitalopram utilization patterns will inform practitioners and policymakers for designing or modifying interventions to improve generic use and increase prescription savings for both patients and payers. This study assessed patterns of and patient factors associated with generic escitalopram initiation and substitution among a large, nationally representative Medicare sample.

## Materials and methods

### Study design and study population

**Fig 1:**
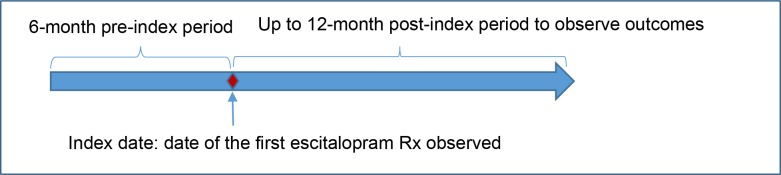
New user cohort study design.

This retrospective, new user cohort study (Fig 1) used a 5% random sample of 2013–2015 Medicare administrative claims data files, which included master beneficiary summary files, Part D prescription drug event files, and Part A (inpatient) and Part B (outpatient) claims files. Fee-for-service Medicare beneficiaries who were continuously enrolled in Parts A, B and D during the 6-month washout period before initiating a brand or generic oral escitalopram prescriptions were included. The date of the initial escitalopram prescription was considered as the index date. All included beneficiaries who initiated brand escitalopram treatment were followed from the index date for up to a 12-month follow up period to observe their generic substitution patterns. Medicare beneficiaries who also enrolled in health maintenance organizations (HMO) or Medicaid any time during the up-to-18 months study period were excluded, as no HMO or Medicaid claims data were available for these beneficiaries. The final study sample included a total of 12,351 escitalopram initiators. All data were fully anonymized by the Centers for Medicare & Medicaid Services (CMS) before we had access. This study was approved by the Auburn University Institutional Review Board and the U.S. FDA Research Involving Human Subjects Committee.

### Outcome measurements

The primary outcome measures were generic oral escitalopram treatment initiation, and among brand oral escitalopram initiators, generic substitution within the 12-month follow up period. National Drug Codes (NDC) of oral escitalopram were used to identify oral generic and brand escitalopram prescriptions via linking NDC in Medicare Part D event files. Escitalopram initiators were categorized based on their initial escitalopram prescription after a 6-month washout period. If the prescription was filled with pills approved through New Drug Application (NDA), the beneficiary was defined as a brand new user. If the prescription was filled with pills approved through ANDA or as an authorized generic (AG), the beneficiary was defined as a generic new user. AG is the same as brand-name drug but is packaged and marketed as generics. Among brand escitalopram initiators, generic substitution was defined as switching from brand product to an AG or generic escitalopram during the 12-month follow up period after index date. Patients who remained on the brand escitalopram treatment until the end of observation were categorized as non-switchers.

In addition, brand escitalopram initiators may encounter other events including switch to alternative treatments, discontinuation, death or loss to follow up, which were considered as competing risks for generic escitalopram substitution. A competing risk event of “switch to alternative treatments” was defined as a brand escitalopram new user switched to an alternative antidepressant treatment [e.g., another SSRI, serotonin–norepinephrine reuptake inhibitors (SNRIs), tricyclic antidepressants (TCAs), monoamine oxidase inhibitors (MAOIs), etc.] during the follow-up period. Treatment discontinuation, as another competing risk event, was defined as a prescription gap in escitalopram or alternative antidepressant treatment exceeding 90 days during the follow up period [16]. Finally, patients who stayed with brand escitalopram treatment until death (using date of death in the Medicare master beneficiary summary files) or loss to follow up (using enrollment data) during the 12-month period after index date were categorized as death/loss to follow up.

### Covariates

To examine factors associated with outcome measures among Medicare beneficiaries, we evaluated a relevant set of beneficiary demographic characteristics, health service utilization factors, and prescription level factors at the pre-index 6-month washout period, index date and follow up period from the Medicare administrative claims data files.

The following factors were identified from the Medicare master beneficiary summary files: age, sex, race, region, end stage renal disease [coded as “yes” if beneficiaries entitled to Medicare based on their end stage renal disease (ESRD)], and Part D low income subsidy (LIS, coded as “yes” if beneficiary had low income cost sharing in any month of the pre-index 6 months washout period). The 5-digit Zip code of each beneficiary was used to match with the 2010 Urban Area to ZIP Code Tabulation Areas (ZCTA) Relationship File [17] to determine a beneficiary’s residence in an urban or rural area.

In addition, beneficiary’s health service utilization factors were identified from Medicare pharmacy, carrier, inpatient and outpatient claims data, including count of unique prescriptions, physician office visits, hospitalization, and emergency department (ED) visits during the pre-index 6 months washout period. Literature indicates that a patient’s previous brand/generic drug use history can influence future brand vs. generic use patterns [18]. Therefore, the proportion of brand drug use was calculated at the beneficiary level and included as a covariate. The calculation was based on the beneficiary’s all prescription use (including refills) during the 6-month pre-index period. The Charlson comorbidity index (CCI) [19] was calculated with included diagnosis codes from Medicare carrier, inpatient, outpatient, skilled nursing home, and home health claims files.

The Medicare Part D event, formulary, and plan files allowed us to identify several prescription/plan level factors based on the initial brand or generic escitalopram prescription on index date including: prescription dispensed as written, prior authorization on formulary, Part D benefit phase (deductible, coverage, gap, catastrophic, other), Part D plan cost sharing in deductible phase, and type of pharmacy for the prescription (community/retail pharmacy, institutional pharmacy, other). Finally, the beneficiary’s escitalopram dosage was examined with both the initial escitalopram prescription and follow up refills prior to the event of studied outcomes.

### Statistical analysis

To identify pre-index factors associated with generic escitalopram treatment initiations, bivariable analyses (Chi-square and t tests) were conducted to compare differences in patient demographic, health service utilization, and prescription level factors between brand and generic escitalopram initiators. Multivariable logistic regression model was used to examine the conditional effects (adjusted odds ratios (OR) and associated 95% confidence intervals (CIs)) of factors on the likelihood of having generic escitalopram treatment initiation, controlling for other covariates. Pearson correlation coefficients were examined among patient and prescription factors to avoid multicollinearity in modeling by dropping selected correlated factors, including counts of prescriptions, ED visits, and physician office visits. In addition, a few covariates (i.e., ESRD, Dispensed as prescribed, and Prior authorization on formulary) were removed due to that the CMS requests no data cell (e.g. admittances, discharges, patients) less than 11 may be published or otherwise displayed [20]. Covariate “Cost sharing in deductible phase” was removed from regression analysis since over 20% patients had missing data (unknown group) for this variable.

Among those who initiated with branded escitalopram, we described patterns of generic substitution, switch to alternative treatments, treatment discontinuation, and death/loss to follow-up during the 12-month follow up period after index date. The Fine-Gray sub-distribution hazards of the cumulative incidence function (CIF) approach [21] were used to estimate cumulative incidence rates (CIRs) of generic substitution and all competing risk outcomes, and CIRs of generic substitution by different patient subgroups (age, sex, race and region). P values of Gray’s test were reported to describe the differences of CIRs [22]. When analyzing data in the presence of competing risks, there are two different types of hazard functions: the cause-specific hazard function and the sub-distribution hazard function. The cause-specific hazard model denotes the instantaneous rate of the primary outcome in those subjects who are currently event free (without competing events), while sub-distribution hazard model allows to directly model the effect of covariates on the incidence of the primary outcome after including competing events [23, 24]. Given that this study focused on the first generic substitution event after the initiation of brand escitalopram treatment, patients who encountered other competing risk events should no longer be at risk for generic substitution. Therefore, the sub-distribution hazard model is not appropriate and we chose the cause-specific hazard function for the competing risk analysis. To evaluate the effects of covariates on time to generic escitalopram substitution among brand initiators, multivariable cause-specific proportional hazards models including time dependent covariate (escitalopram daily dosage) were fitted [25]. Additional analyses to estimate factors associated with rates of generic substitution were also conducted to confirm the robustness of main findings: 1) multivariable cause-specific proportional hazards models without time dependent dosage, 2) Fine-Gray competing risk models with time dependent dosage, and 3) Fine-Gray competing risk models without time dependent dosage, respectively. All analyses were conducted using SAS (version 9.4; SAS Institute, Inc., Cary, NC). Statistical significance was set at P<0.05.

## Results

### Generic escitalopram initiation

In 2013–2015, among the 12,351 Medicare escitalopram initiators, there were 10,887 (88.2%) beneficiaries who began with generic treatment and the rest (11.8%) initiated brand treatment (Table 1). Results from bivariable analyses showed that, compared to beneficiaries who initiated branded escitalopram, generic initiators tended to be younger (<75), male, and residing in the Midwest region and/or urban areas (all P<0.05). In addition, more beneficiaries who initiated generic escitalopram used fewer medications, had fewer physician office visits, and used a lower proportion of brand name drugs during the pre-index 6-month period. However, more generic initiators had a higher mean initial daily dose and were more likely to be enrolled in the deductible Part D plan benefit phase, have cost sharing in deductible phase, and fill their initial escitalopram prescription in community/retail pharmacies (all P<0.05). In the multivariable logistic regression analysis adjusting for all covariates, the remaining statistically significant independent factors associated with higher likelihood of generic escitalopram initiation were: younger age, male sex, residence in non-Northeast regions and urban area, enrollment in deductible Part D plan benefit phase, and filling prescription at community/retail pharmacies (all P<0.05, Table 1).

**Table 1:** Sample characteristics and factors associated with generic escitalopram initiation (n = 12,351).

Factors	Generic Initiation		Brand Initiation		P^a^	Generic Initiation ^b^
	N	%	N	%		AOR (95% CI)
**Sample size**	10887	88.2	1464	11.8		
**Patient characteristics**						
**Age**					**< .001**	
<65	1377	12.7	151	10.3		Ref
65–74	4366	40.1	539	36.8		0.89 (0.72,1.10)
75–84	3238	29.7	476	32.5		**0.78 (0.62,0.97)**
85 and older	1906	17.5	298	20.4		**0.77 (0.61,0.98)**
**Sex**					**0.03**	
Male	3438	31.6	420	28.7		Ref
Female	7449	68.4	1044	71.3		**0.87 (0.77,0.98)**
**Race/ethnicity**					0.68	
White	10065	92.5	1362	93.0		Ref
Black	483	4.4	58	4.0		1.09 (0.82,1.46)
Others ^c^	339	3.1	44	3.0		1.00 (0.72,1.38)
**Region**					**< .001**	
Northeast	2107	19.4	380	26.0		Ref
Midwest	2770	25.4	257	17.5		**2.01 (1.69,2.38)**
South	4408	40.5	632	43.2		**1.27 (1.11,1.46)**
West	1602	14.7	195	13.3		**1.46 (1.21,1.76)**
**Urban vs. rural**					**0.032**	
Urban	9659	88.7	1271	86.8		**1.29 (1.09,1.52)**
Rural	1228	11.3	193	13.2		Ref
**LIS eligible**					0.74	
Yes	1534	14.1	211	14.4		0.95 (0.79,1.14)
No	9353	85.9	1253	85.6		Ref
**Patient health service utilization factors at pre-index period**						
**Count of unique prescriptions (mean, SD)**	Mean: 8.8	Sd:5.0	Mean: 9.1	Sd: 4.9	**0.048** **0.01**	
1	313	2.9	31	2.1		N/A
2–5	2775	25.5	327	22.3		
6–10	4370	40.1	606	41.4		
11 or more	3429	31.5	500	34.2		
**Count of hospitalizations (mean, SD)**	Mean: 0.46	Sd: 0.97	Mean: 0.47	Sd: 0.98	0.09 0.36	
0	8011	73.6	1064	72.7		Ref
1	1648	15.1	242	16.5		0.95 (0.79,1.14)
2 or more	1228	11.3	158	10.8		1.15 (0.94,1.41)
**Count of ED visits (mean, SD)**	Mean: 0.77	Sd: 1.49	Mean: 0.78	Sd: 1.37	0.76 0.50	
0	6691	61.5	880	60.1		N/A
1	2236	20.5	305	20.8		
2–3	1461	13.4	216	14.8		
4 or more	499	4.6	63	4.3		
**Count of physician office visits (mean, SD)**	Mean: 22.2	Sd: 24.5	Mean: 23.3	Sd: 23.9	0.09 **0.01**	
1 or less	487	4.5	61	4.2		N/A
2–10	3543	32.5	414	28.3		
11–20	2832	26.0	413	28.2		
21–30	1567	14.4	211	14.4		
31 or more	2458	22.5	365	24.9		
**Charlson comorbidity index (mean, SD)**	Mean: 2.3	Sd: 2.7	Mean: 2.4	Sd: 2.6	0.24 0.07	
0	3322	30.5	394	26.9		Ref
1	2262	20.8	310	21.2		0.90 (0.76,1.05)
2	1595	14.6	223	15.2		0.90 (0.75,1.08)
3	1188	10.9	176	12.0		0.86 (0.70,1.05)
4 or more	2520	23.2	361	24.7		0.88 (0.73,1.05)
**Proportion of brand drug use**	Mean: 0.16	Sd:0.18	Mean: 0.17	Sd:0.19	**0.02** **0.03**	
0	3789	34.8	465	31.8		1.04 (0.90,1.19)
0.001–0.199	3541	32.5	476	32.5		Ref
0.20 or more	3557	32.7	523	35.7		0.92 (0.81,1.06)
**Prescription-level factors**						
**Daily dosage (mean, SD)**	Mean: 10.7	Sd: 6.4	Mean: 10.3	Sd: 4.6	**0.003**	1.01 (0.999,1.02)
**Part D plan benefit phase**					**0.01**	
Deductible	1057	9.7	109	7.4		Ref
Coverage	6006	55.2	849	58.0		**0.80 (0.65,0.998)**
Gap	1137	10.4	154	10.5		0.91 (0.69,1.20)
Catastrophic	331	3.1	57	3.9		**0.68 (0.47,0.98)**
Others ^d^	2356	21.6	295	20.2		0.97 (0.76,1.24)
**Cost sharing in deductible phase**					**0.03**	
Yes	524	4.8	52	3.5		N/A
No	8006	73.5	1117	76.3		
Unknown	2357	21.7	295	20.2		
**Type of pharmacy**					**< .001**	
Community/retail pharmacy	8845	81.2	1097	74.9		Ref
Institutional pharmacy	981	9.0	177	12.1		**0.74 (0.61,0.90)**
Others ^e^	1061	9.8	190	13.0		**0.69 (0.58,0.81)**

Abbreviation: LIS: Low-income subsidy; N/A: not applicable (either due to small cell size or excluded from multivariable analysis because of covariates correlations).

^a^ Chi-square or t test.

^b^ Multivariable logistic regression model to identify factors associated with generic treatment initiation; adjusted odds ratios (AOR) and 95% confidence intervals (CI) reported.

^c^ Others, including Asian, Hispanic, North American Native, Unknown, all the rest and missing.

^d^ Others, including beneficiary enrolled in PACE or employer-sponsored plan, all the rest and missing.

^e^ Others, including mail order pharmacy, specialty care pharmacy, all the rest and missing.

### Generic escitalopram substitution

**Fig 2:**
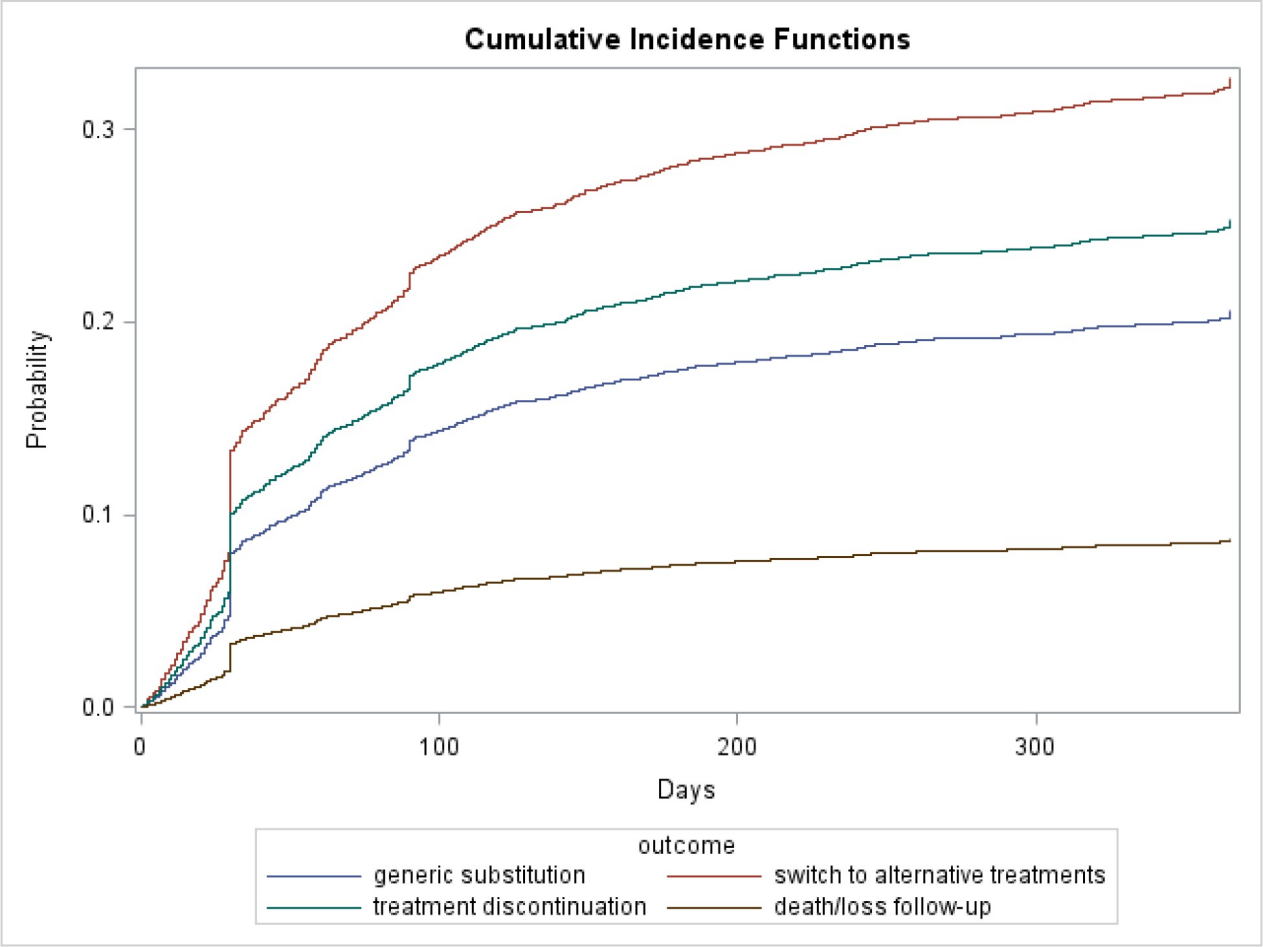
Cumulative incidence function plots for time to generic substitution and all competing risk outcomes within 12 months among Medicare beneficiaries who initiated brand escitalopram treatment (n = 1,464).

**Fig 3:**
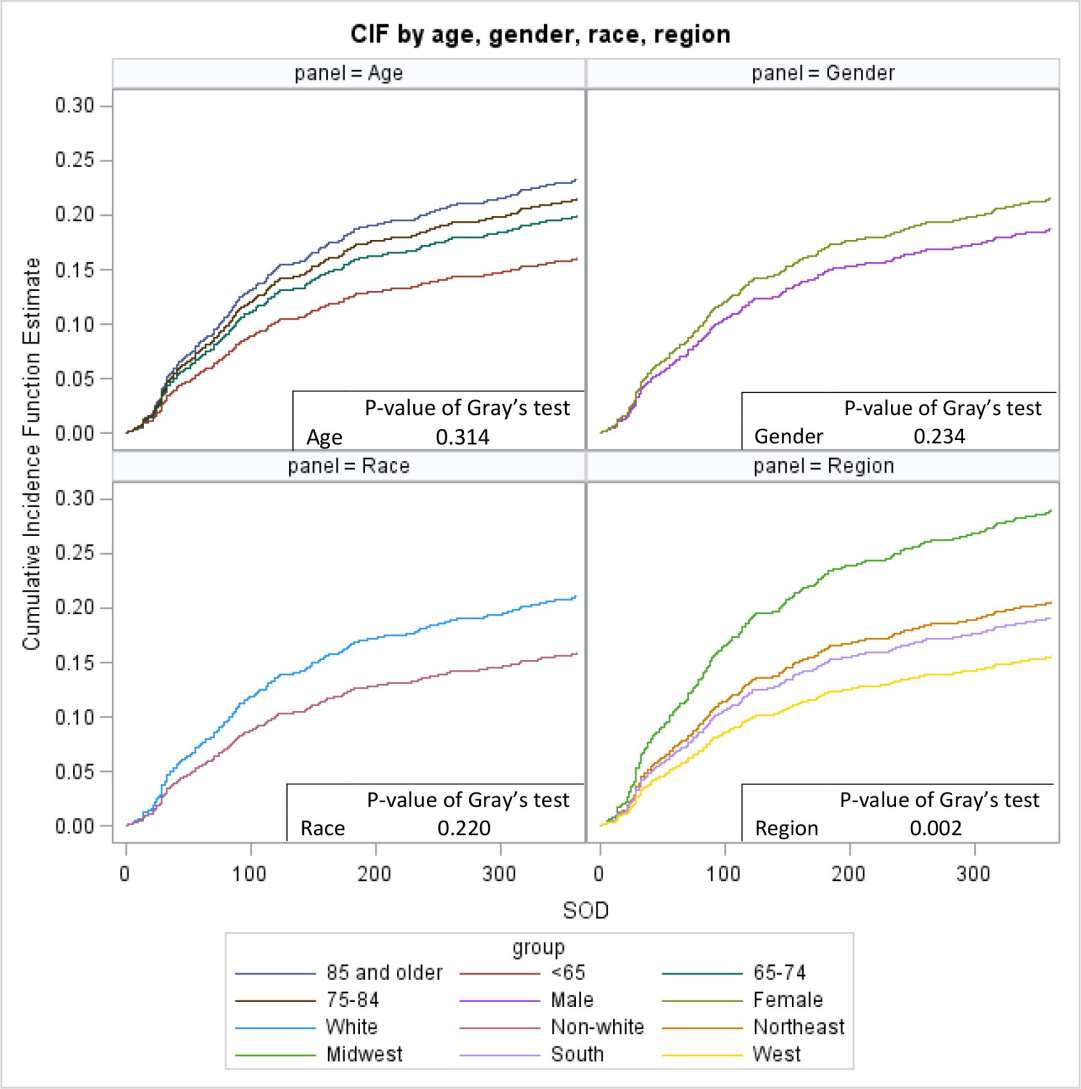
Cumulative incidence function plots for time to generic substitution within 12 months by different patient subgroups (age, sex, race and region) among Medicare beneficiaries who initiated brand escitalopram treatment (n = 1,464).

Among Medicare beneficiaries who initiated brand escitalopram (n = 1,464), results from unadjusted CIF estimates (Fig 2) indicated that, by the end of 12 months after treatment initiation, the CIRs for outcomes were different. Specifically, the CIR for generic escitalopram substitution was 20.7%. The CIR was 31.2% for beneficiaries who switched to alternative antidepressant treatments, 25.1% for those who discontinued treatments, and 8.7% for beneficiaries who passed away or were lost to follow-up. In addition, CIRs for generic substitution by different patient subgroups (Fig 3) showed that there were no significant differences for generic escitalopram substitution across age, sex, race and region subgroups, except for patients in the Midwest region who switched much faster and in higher proportion than other regions (P = 0.002).

**Fig 4:**
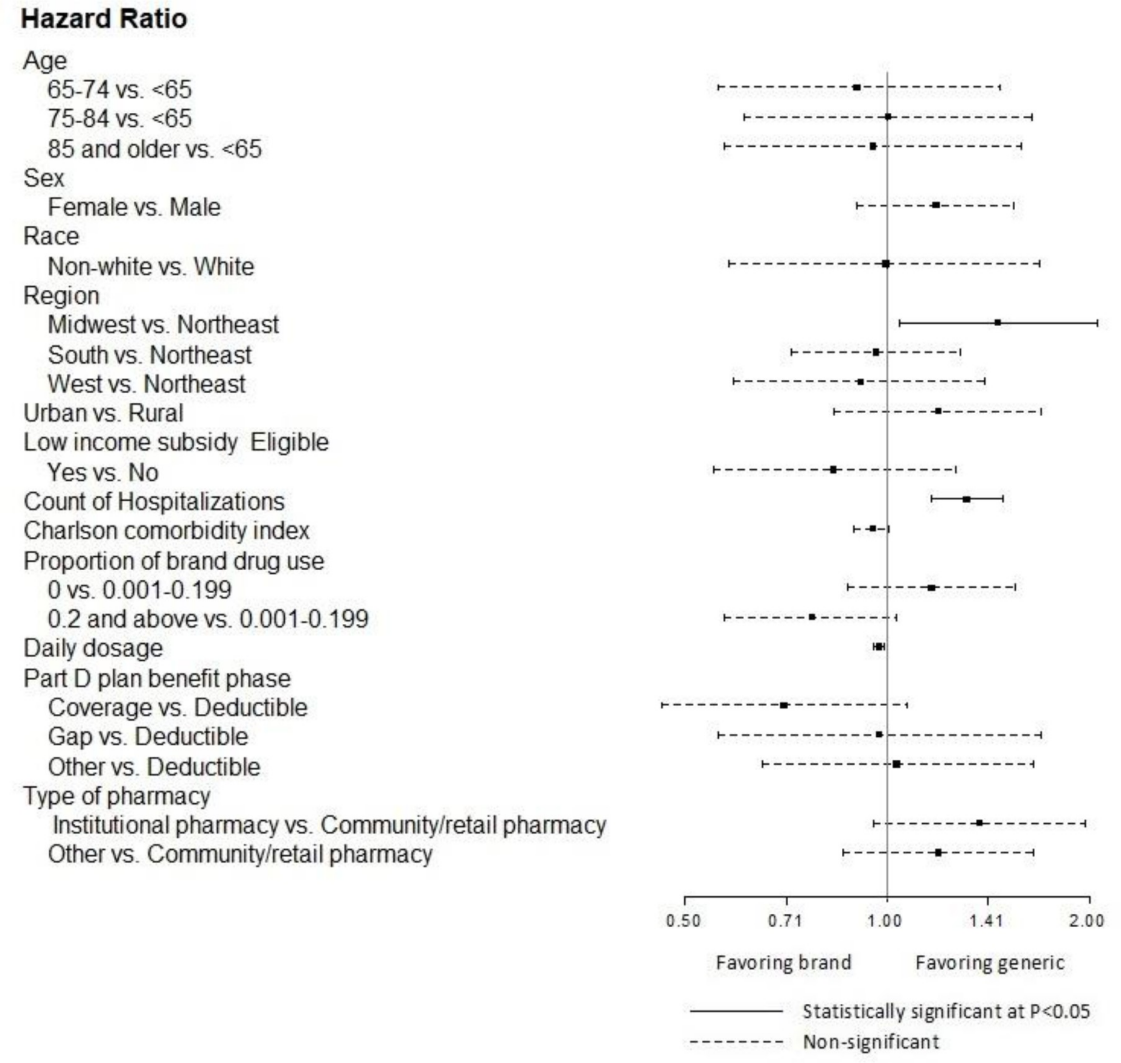
Results from cause-specific proportional hazards model of time to generic substitution within 12 months among brand escitalopram initiators (adjusted hazard ratios and 95% CI).

In the multivariable model of time to generic substitution, a few significant patient or prescription factors were associated with rate of generic substitution (Fig 4). Specifically, those who resided in the Midwest region had higher rates of generic substitution (cause-specific hazard ratio (HR) = 1.46 vs. Northeast; 95% CI = 1.04–2.05). An additional episode of prior hospitalization was associated with a 31% increase in rate of generic substitution (HR = 1.31, 95% CI = 1.16–1.48), and an additional unit of prior daily dosage was associated with a statistically significant 3% decrease in rate of generic substitution (HR = 0.97; 95% CI = 0.95–0.99). Sensitivity analyses using Fine-Gray competing risk models or initial dosage instead of time dependent dosage demonstrated similar results.

## Discussion

In this retrospective new user cohort of nationally representative Medicare escitalopram initiators, we found that almost 90% of patients initiated with generic, rather than brand, treatment in 2013–2015. This indicates that generic escitalopram treatment was well accepted by Medicare Part D prescribers and beneficiaries one year after the initial generic escitalopram approval from the FDA. In 2013, about 84% of all prescriptions were filled with generics in the U.S. [26], and it increased to 90% in 2017 [12]. Generic escitalopram initiation and substitution provides significant savings in prescription spending to the U.S. healthcare system (for example, $11 for 30 tablets of generic escitalopram 10mg vs. $330 for 30 tablets of brand Lexapro® 10mg, according to goodrx.com in August, 2018). These savings were made possible by the faster user-fee funded FDA generic drug approval process [27], state generic substitution laws [28], the Protecting Consumer Access to Generic Drugs Acts [29, 30], and increased healthcare professional [16, 31, 32] and patient [33, 34] acceptance and knowledge of generics.

Regarding patient and prescription factors associated with generic escitalopram initiation, we found that Medicare beneficiaries were less likely to initiate generic treatment if they were 75 years old or older, female, residing in the Northeast region, or residing in a rural area. These findings suggested that patients’ demographic characteristics, rather than health service utilization, could have a significant impact on their generic escitalopram treatment initiation. It further highlighted that the decision making in brand or generic escitalopram treatment initiation may be impacted by patients’ and providers’ preference or access of generic drugs. In the past, studies found that older patients had negative attitudes and beliefs about generic medications [35, 36]. Other qualitative studies among female Medicaid beneficiaries [37] and patients in rural area with lower socioeconomic status [38] reported mistrust and lower preference in generic drugs. Policymakers can target patient’s demographic factors identified from this study to tailor and launch educational outreach programs for improving patient’s knowledge and acceptance regarding generic drugs and further increasing generic drug use among those subgroup populations.

Among Medicare beneficiaries who initiated brand escitalopram treatment, we observed that 1 in 5 initiators encountered generic substitution within 12 months. However, higher proportions of brand initiators switched to an alternative antidepressant (31.2%) or discontinued (25.1%) treatment than those who continued escitalopram treatment with a generic (20.7%) or stayed on brand treatment (14.9%). The higher rates of switching to alternative treatments or discontinuation may be explained by escitalopram’s overall response rate of around 48%-62% for patients with major depressive disorder [39, 40] or social anxiety disorder [41], as indicated by a few systematic review and meta-analysis studies. Studies also found that drug-related adverse effects such as intolerability were the most frequent reason for switching or discontinuing SSRIs within the first 3 months of treatment [42, 43]. In addition, clinicians also consider switching as a strategy that involves substitution of another antidepressant drug for current treatment that is either lack of a response or intolerable, which could also help explain the higher switching rate found in the current study [44, 45]. A newly published guideline review demonstrated that about half of patients with depression require second-line treatment to achieve remission, but consistency and clarity in current guidelines for second-line treatment of depression are lacking [46]. In addition, Ereshefsky et al. studied initiators of brand escitalopram and found that only 20.4% of patients stayed on escitalopram treatment, and 73.4% discontinued after 6 months [7]. We were unable to identify the direct reasons for treatment discontinuation due to lack of data access to electronic health records (EHR) data. However, possible reasons for higher discontinuation rate and lower persistent use rates for antidepressant treatments have been studied extensively and attributed to patients’ poor drug adherence [47], side effects [48, 49], concerns with medication tolerability [50], and increased healthcare costs [6, 7, 51]. Timely communication between patients and providers is a key factor for optimizing treatment outcomes for patients who require antidepressant treatment.

We observed geographic differences in generic escitalopram initiation and substitution among Medicare beneficiaries. Specifically, beneficiaries in the Midwest, West and South regions were more likely to encounter generic initiation than those in the Northeast. After controlling for various patient demographic factors, health service utilization, and prescription level factors, brand escitalopram initiators in the Midwest were more likely to substitute with generics than those in the Northeast region. One explanation of the geographic difference in generic and brand escitalopram use may be healthcare provider prescribing behaviors. The key findings of the Express Scripts 2012 Drug Trend Report were that providers in Midwestern states such as Ohio, Illinois and Michigan prescribed generics more often than Northeastern states (such as New York and New Jersey) and some Southern states (e.g. Texas and Louisiana) [52]. One of our previous publications also found that Medicare Part D prescribers in the Midwest, West and South regions had higher annual generic drug prescribing rates than those in the Northeast [53]. Evidence suggests that multiple factors, such as physicians' knowledge and values [54], financial relationship with pharmaceutical industries [53], and patient’s health insurance [55], can impact prescribing behaviors. Therefore, educational outreach regarding generic drugs that targets prescribers, patients, policymakers, and formulary managers can be considered to reduce geographic differences in generic drug use, increase patient’s access to generics, and reduce prescription spending for both patients and payers.

Finally, we found that patient’s pre-index hospitalizations (positively) and their escitalopram daily dosage (negatively) were associated with rate of generic substitution. Patient’s number of pre-index hospitalizations can reflect their overall health status. That is, patients with prior hospitalizations might be in worse health status and have higher economic burden on health services including prescriptions. In contrast, escitalopram daily dosage might correlate with a patient’s disease severity and response to treatment [56]. According to the American Psychiatric Association’s practice guidelines for treatment of major depressive disorders, once an antidepressant treatment has been initiated, the titration to full therapeutic dose should depend upon many factors such as patient’s age, treatment setting, presence of comorbidities, concomitant pharmacotherapy, and/or treatment side effects [57]. When side effects occur, an initial strategy is to lower the dose of the antidepressant or to change to an antidepressant that is not associated with that side effect [57]. Therefore, the negative association between escitalopram dosage and generic substitution found in our study may reflect the case of occurrence of side effects prior to generic substitution decisions. Future research needs to investigate how physicians’ decision making in generic escitalopram substitution is impacted by patient health status and their experience with treatment-associated side effects.

This study has a few limitations. First, although the new user design strengthens the observational study design by eliminating more confounders, study findings imply associations rather than causality. Second, patients’ perceptions and prescribers’ characteristics were not assessed due to lack of data access or missing data (i.e. National Provider Identifier in Medicare claims data). Third, it is unable to verify whether patients with dispensed medications actually took the medications, which is a strong assumption especially for patients with mental health disorders. Fourth, 6 months instead of 12 months was used for the pre-index washout period, in which seasonality on sample admissions to the new user cohort may have skewed the distribution of the covariates. Fifth, this study only included Medicare beneficiaries, so findings may not be generalizable to other non-Medicare populations. Finally, this analysis only examined patients’ first follow-up events after brand escitalopram initiation. Other events, such as switching back to brand treatment, acute clinical outcomes such as hospitalization, or disease episode relapse, warrant for further studies.

## Conclusions

In conclusion, almost 90% Medicare beneficiaries initiated generic escitalopram treatment in 2013–2015. Among brand escitalopram initiators, the 1-year generic substitution rate was around 20%. Beneficiary’s age and sex were associated with generic escitalopram initiation. Beneficiary’s geographic region was independently associated with generic escitalopram initiation and substitution. Findings provide evidence of recent generic escitalopram use patterns among the Medicare population and can guide educational and practice interventions to further increase generic escitalopram use.
